# Labial Gland Mesenchymal Stem Cell Derived Exosomes-Mediated miRNA-125b Attenuates Experimental Sjogren’s Syndrome by Targeting PRDM1 and Suppressing Plasma Cells

**DOI:** 10.3389/fimmu.2022.871096

**Published:** 2022-04-04

**Authors:** Yixiao Xing, Boya Li, Jing He, Hong Hua

**Affiliations:** ^1^ Department of Oral Medicine, Peking University School and Hospital of Stomatology, Beijing, China; ^2^ Central Laboratory, Peking University School and Hospital of Stomatology, Beijing, China; ^3^ Department of Oral Medicine, First Hospital Affiliated to Zhengzhou University, Zhengzhou, China; ^4^ Department of Rheumatology and Immunology, Peking University People’s Hospital, Beijing, China

**Keywords:** Sjogren’s syndrome, B cells, plasma cells, mesenchymal stem cells, immunomodulation, labial gland

## Abstract

The pathogenesis of the prototypical chronic autoimmune disorder primary Sjögren syndrome (pSS) has been thought to be B-cell-centric, based on serum autoantibodies, the increased risk of B cell lymphoma, and altered B cell subsets in patients with pSS. Over the last 10 years, therapies targeting B cells have been investigated for pSS; however, current evidence for the efficacy of B cell targeted therapies in pSS is still sparse. Mesenchymal stem cells (MSCs) might represent a promising strategy for cell therapy of autoimmune diseases *via* regulation of immune cells. MSC-released exosomes carry various bioactive molecules and thus have been studied in MSC-based therapy. The newly discovered labial gland MSCs (LGMSCs) have exhibited enhanced performance. Herein, we aimed to determine the effects of LGMSC-derived exosomes (LGMSC-Exos) on the symptoms of a mouse model of pSS and their regulatory effect and mechanism on B cell subsets. *In vivo*, treatment of the spontaneous mouse model of pSS with LGMSC-Exos resulted in reduced inflammatory infiltration and restored saliva secretion in salivary glands. *In vitro*, coculture of LGMSC-Exos with peripheral blood mononuclear cells of patients with pSS markedly reduced the proportions of CD19^+^CD20^-^CD27^+^CD38^+^ plasma cells among peripheral blood mononuclear cells. Further investigations provided evidence that LGMSC-Exo-derived microRNA-125b affected plasma cells of pSS by directly binding to its target gene, *PRDM1* (PR domain zinc finger protein 1, also known as BLIMP1), which might be developed as a target to treat pSS. Overall, these findings provided a possible exploitable therapeutic target in pSS and provide new insights into the potential therapeutic application of exosomes in pSS and other disease mediated by B-cells.

## Introduction

The systemic autoimmune disease, primary Sjögren’s syndrome (pSS), is characterized by exocrinopathy, often causing dryness of the eyes and mouth, fatigue, and joint pain (1). In addition to glandular manifestations, 33-50% of patients suffer from systemic involvement (2). In addition, the most severe complication of pSS is non-Hodgkin lymphoma, which leads to a worse prognosis in 5–10% of patients (3). pSS is estimated to occur in 0.3–3 per 1,000 of the whole population (4), posing a significant burden to patient quality of life and health systems.

Although the pathogenesis of the disease remains obscure, abnormalities or hyperactivation of T cells and B cells have been suggested to play an important role. Recent evidence indicated that in pSS pathogenesis, B cells have a major and vital function (5). B cell hyperactivity in patients with pSS is revealed by a number of biological signs, such as increased levels of serum free light chains and the presence of anti-Sjögren-syndrome-related antigen A (SSA; also known as Ro) antibodies, anti-Sjögren-syndrome- related antigen B (SSB; also known as La) antibodies, and rheumatoid factor (RF). In contrast to systemic lupus erythematosus and rheumatoid arthritis, pSS-specific auto-antibodies can appear before the emergence of symptoms, suggesting that B cells have a vital early function in pSS (6). Additionally, pSS target organs, such as salivary glands, contain B cells that sometimes form structures resembling germinal centers, which might lead to a higher risk of developing lymphoma (7). Patients with pSS have a higher risk of developing B cell lymphoma compared with both patients with other rheumatic diseases, like rheumatoid arthritis and systemic lupus erythematosus, and the general population (8), again illustrating the role of B cells in pSS pathogenesis.

Consequently, therapies designed to reduce the B cell population or inhibit B cell activation have been evaluated (9). Several B cell molecules might be targeted, such as CD20 (also known as membrane spanning 4-Domains A1), CD22, B-cell-activating factor (BAFF), and BAFF receptors. Among them, to achieve B-cell depletion, CD20 is the most widely studied target. Although early, small-scale studies showed promising results, two recent large randomized controlled trials of Rituximab to treat pSS did not meet their primary endpoints (10, 11). Nevertheless, Rituximab does impact the underlying disease process by ameliorating B-cell hyperactivity and glandular histology in some patients (12). The efficacy of an anti-BAFF monoclonal antibody, belimumab, was evaluated in the BELISS open label prospective phase II trial. The results seemed to be promising in pSS (13); however, its efficacy needs to be evaluated in larger clinical trials. For patients with pSS, depleting B-cells seems to be a promising treatment strategy; however, this depletion is non-selective and the effects on exocrine function and sicca symptoms remained limited.

Mesenchymal stem cells (MSCs) are heterogeneous progenitor cells that can be isolated from a variety of sources. Meaningful benefits of MSCs-based therapy in rheumatology, especially in refractory cases, have been demonstrated (14) (15). MSCs react with immune cells and exert their immunoregulation effect by reprogramming immune cells and reducing inflammation *via* secreted paracrine factors (16) (17). MSCs produce extracellular vehicles, including micro vesicles and exosomes, which are believed to be the main bioactive vesicles that mediate MSC’s paracrine effects and are effectors of cell signaling and cell-to-cell communication, thus playing a variety of roles, including immune regulation (18, 19). Compared with their parental cells, MSC-derived exosomes are easier to store and transport, and are of less concern regarding embolism and tumorigenicity. Therefore, MSC-derived exosomes are being exploited for cell-free therapies to replace cell-based MSC therapies.

MSCs derived from labial gland (LGMSCs) were first isolated by our group (20) and we showed that they exhibit characteristic MSCs properties. Therefore, the present study aimed to assess the immunoregulatory effect of exosomes from LGMSCs on B cells in a mouse pSS model and in patients with pSS *in vitro*. We further evaluated the possible mechanism of LGMSC exosomes on B cell subsets by examining the genes involved in the differentiation and activation of B cells and the microRNA (miRNA) responsible for the exosomes’ effect.

## Materials And Methods

### Mice

The Guidelines of Peking University Institutional Review Board for the care and use of laboratory animals were followed for the animal experiments. The Laboratory Animal Center of Peking Medical University provided the female BALB/c and non-obese diabetic (NOD) mice (both 6–8 weeks old). Mice were housed in a specific pathogen-free environment and had *ad libitum* access to standard rodent chow and water.

### Ethics Statement

The institutional review board of Peking University School of Stomatology approved the collection of human peripheral blood and human labial glands for research purposes (PKUSSIRB-201631139). Samples were collected after obtaining informed written consent from the patients.

### LGMSC Culture, Isolation, and Characterization

Samples from five donors who required surgery to remove their lip mucoceles, and were free from any systemic diseases, were used to isolate LGMSCs. The adjacent normal labial glands (LGs) were collected after enucleation of lip mucoceles. After isolation, phosphate-buffered saline (PBS) (Gibco, Waltham, MA, USA) was used to wash the LGs and their surrounding fibrous tissues and blood vessels were carefully wiped away. Then, the remaining tissues were cut into small parts and cultured in cell culture flasks containing alpha-Minimum Essential Medium (α-MEM) (Gibco) with 20% fetal bovine serum (Gibco) and 1% penicillin/streptomycin (Beyotime, Shanghai, China) in a humidified atmosphere of 5% CO_2_ at 37°C. When the cells reached 80%–90% confluence, 0.25% trypsin (MP Biomedicals, USA) was used to digest the adherent cells, which were passaged *in vitro*. All the experiments used MSCs at the third and fourth passages.

LGMSCs were identified using previously described methods (21), including surface marker assessment using flow cytometry, osteogenic differentiation, and adipogenic differentiation.

### Isolation and Identification of Exosomes Derived From LGMSCs (LGMSC-Exos)

LGMSCs were grown in alpha-Minimum Essential Medium containing 10% exosome-depleted fetal bovine serum for 48 h, after which we collected the culture supernatants. The supernatants were centrifuged sequentially at 300 × *g* for 10 min, 2000 × *g* for 10 min, and 10,000 × *g* for 30 min. Lastly, the supernatants were subjected to ultracentrifugation at 100,000 × g at 4°C for 60 min using an Optima L-100XP ultracentrifuge (Beckman Coulter, Placentia, CA, USA), followed by washing using PBS and centrifugation at 100,000 × *g* for another 1 h. A BCA Protein Assay Kit (Thermo Fisher, Waltham, MA, USA) was used to determine the protein concentration to quantify the LGMSC-Exos. Nanoparticle tracking analysis (NTA), transmission electron microscopy (TEM) observation, and western blotting were used to characterize the purified LGMSC-Exos.

For western blotting, we used anti-CD63 and CD81 antibodies (1:1000 dilution; (Abcam, Cambridge, UK) (Abcam). For NTA, exosomes were diluted to a suitable concentration with PBS and analyzed using a ZetaView PMX 110 instrument (Particle Metrix, Munich, Germany). For TEM observation, the concentration of the exosomes was adjusted to roughly 10^11^ vesicles per mL using PBS and 20 μL of the diluted exosomes were deposited on copper coated 200-mesh formvar grids and dried for 5 min at room temperature. Samples were then stained with 10 μL phosphotungstic acid for 2 min at room temperature. Filter paper was used to blot off the excess solution, and the samples were dried at room temperature, observed under a transmission electron microscope, and photographed.

### Isolation of Human Peripheral Blood Mononuclear Cells (PBMCs) and Their Co-Culture With LGMSC-Exos or LGMSCs

Peripheral blood from patients with pSS or age- and sex- matched donors was collected into EDTA Vacutainers. Ficoll density-gradient centrifugation (Haoyang Biotech, Tianjin, China) was used to isolate PBMCs. In co-culture system, the LGMSCs and PBMCs were added at a ratio of 1:10, and exosomes (30 μg/mL) were added into complete Roswell Park Memorial Institute 1640 medium for PBMCs treatment. PBMCs were collected for subsequent experiments at 72 h after co-culture. To evaluate whether effect of LGMSCs on B cells is mediated by their exosomes, GW4869, a commonly used pharmacological agent that inhibits exosome generation was used. LGMSCs and PBMCs were cultured in regular culture medium with 10 μM GW4869. To evaluate PRDM1/Blimp1 expression in B cells after treated with LGMSC-Exos, PBMCs were collected at 72 h after co-culture and then MagniSortTM Human CD19 Positive Selection Kit (Thermo Scientific, MA, USA) was used to isolate CD19^+^ B cells from PBMCs.

### Mice Intervention

All mice used were female. NOD mice transplanted with LGMSCs or LGMSC-Exos comprised the treatment groups. NOD mice intragastrically administrated with hydroxychloroquine (HCQ) served as the positive controls and mice infused with PBS comprised the negative controls. For transplantation, LGMSCs (1 × 10^6^ diluted in 200 μL PBS/mouse) or LGMSC-Exos (50 μg diluted in 200 μL PBS/mouse) were injected into NOD mice *via* their tail veins on alternate days for 14 days. Body weight, serum glucose levels, and the salivary flow rate of the mice were measured every other week until mice were sacrificed at week 16.

### Flow Cytometry Analysis

PBMCs co-cultured with LGMSCs or LGMSC-Exos and splenic lymphocytes collected after the mice were sacrificed were subjected to flow cytometry analysis for B cell subsets. For PBMCs, they were incubated with anti-human CD19, CD20, IgD, CD38, CD27, and CD24 monoclonal antibodies (5 μL per million cells in a 100 μL staining volume) (BioLegend, San Diego, CA, USA) for 30 mins. For mouse splenic lymphocytes, they were incubated with anti-mouse CD19, CD27, and CD138 monoclonal antibodies (5 μL per million cells in a 100 μL staining volume) (BioLegend) for 30 mins. Thereafter, the cells were washed two times before being analyzed on a CytoFLEX flow cytometer (Beckman Coulter, Brea, CA, USA).

### RNA Isolation and Quantitative Real-Time Reverse Transcription PCR (qRT-PCR)

The Trizol Reagent (Takara, Tokyo, Japan) was used to extract total RNA from cells according to the supplier’s instructions. A PrimeScript™ RT Reagent Kit (Takara) was used to synthesize cDNA from the RNA. cDNA representing miRNA was generated using a miRNA First Strand cDNA Synthesis Kit (Sangon Biotech, Shanghai, China). The quantitative real-time PCR step of the qRT-PCR protocol was performed in a reaction volume of 10 μL using SYBR^®^ Premix Ex Taq™ by Roche LightCycler^®^ 480II (Roche, Mannheim, Germany). The relative expression levels of genes were calculated using the 2^–ΔΔCT^ method (22), and the primer sequences are listed in **Table 1**.

**Table 1:** Sequences of primers.

Genes or miRNAs		Sequences
PRDM1	Forward (5ˊ~3ˊ)	AAGATCAAGTACGAATGCAACG
	Reverse (5ˊ~3ˊ)	TGCAAGTCTGACATTTGAAAGG
GAPDH	Forward (5ˊ~3ˊ)	GCACCGTCAAGGCTGAGAAC
	Reverse (5ˊ~3ˊ)	TGGTGAAGACGCCAGTGGA
miRNA-125b-5p F	Forward (5ˊ~3ˊ)	CTCCCTGAGACCCTAACTTGTGA
Universal U6 Primer	Forward (5ˊ~3ˊ)	miRNA First Strand cDNA Synthesis Kit
Universal PCR Primer R for miRNA	Reverse (5ˊ~3ˊ)	miRNA First Strand cDNA Synthesis Kit

### Dual Luciferase Reporter Assay

The interaction between *PRDM1* (encoding PR domain zinc finger protein 1) and hsa-miR-125b-5p (miR-125b) was predicted using TargetScan software (version 7.1) (23). Position 166–172 of the *PRDM1* 3’ UTR was predicted to bind to miR-125b. The *PRDM1* fragment containing the miR-125b binding sites was synthesized to generate wild-type (PRDM1–WT) or mutant-type (PRDM1–MUT). The reporter of fluorescence in the vector was encoded by the Renilla Luciferase gene (Rluc), and PRDM1–WT and PRDM1–MUT were cloned downstream of Rluc the gene, separately. Then, the luciferase reporter plasmids PRDM1–WT and PRDM1–MUT (RiboBio, Guangzhou, China) were co-transfected with miR-125b mimic or control mimics (RiboBio) into HEK-293 T cells. Forty-eight hours after transfection, the Glo^®^ Luciferase Assay System (Promega, Madison, WI, USA) was used to lyse the cells and a GLomax20/20 Luminometer (Promega) was used to determine the luciferase activity.

### Depletion and Upregulation of miR-125b in LGMSC-Exos

MiR-125b in exosomes was depleted and upregulated by transfection of miR-125b mimics and inhibitors (RiboBio), respectively, into LGMSC-Exos using a Fect™ Exosome Transfection Kit (System Biosciences, Palo Alto, CA, USA).

### Statistical Analysis

Data are presented as the mean ± standard deviation. The statistical analyses were carried out using GraphPad Prism version 6.01 (GraphPad Software, La Jolla, CA, USA) or IBM SPSS Statistics version 26 (IBM, Armonk, NY, USA). Differences between groups were analyzed using the Mann–Whitney U-test or Kruskal–Wallis comparison test.

## Results

### Characterization of LGMSCs and LGMSC-Exos

**Figure 1:**
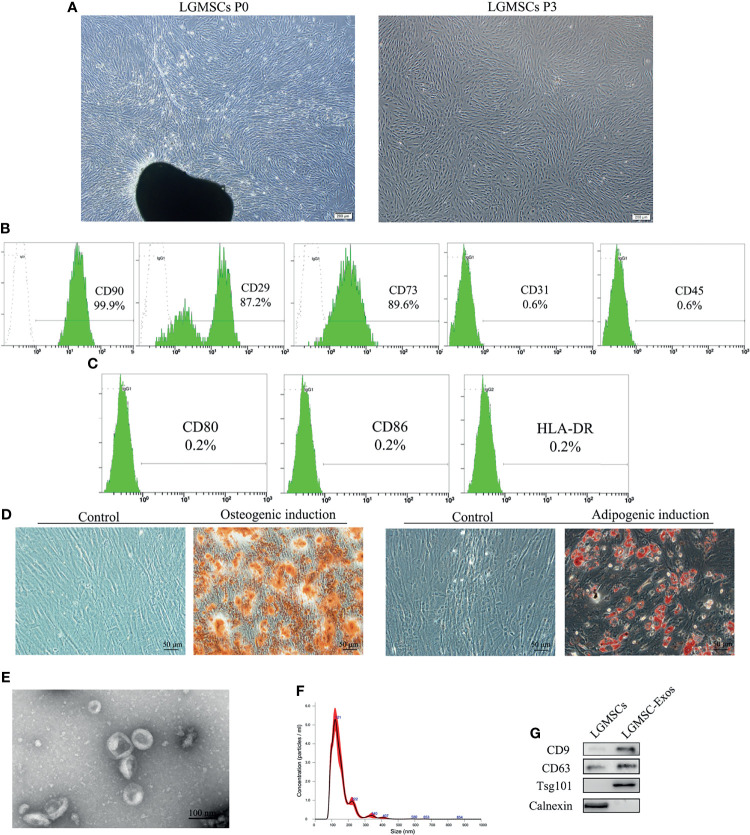
Isolation and identification of LGMSCs and LGMSC-derived exosomes. **(A)** LGMSCs at passage 0 (P0) and P3; **(B)** Flow cytometry results of surface markers of LGMSCs; **(C)** Flow cytometry results of costimulatory molecule expression on LGMSCs; **(D)** osteogenic and adipogenic induced LGMSCs stained using Alizarin red staining and oil red O; **(E)** Representative TEM (bar = 100 nm) micrographs of LGMSC-Exos; **(F)** particle size of LGMSC-Exos analyzed using NTA; **(G)** Western blotting analysis of CD9, CD63, Tsg101, and calnexin in LGMSCs and LGMSC-Exos.

LGMSCs exhibited a typical spindle-shaped morphology (**Figure 1A**) and expressed the MSC markers CD29, CD73, and CD90 (99.9%), but did not express the endothelial cell marker CD31 or the hematopoietic marker CD45 (**Figure 1B**). Moreover, costimulatory molecule expression (HLA-DR, CD86, and CD80) was negative (**Figure 1C**). Their multiple differentiation potential was demonstrated after induction for 21 days using alizarin red staining (for osteogenesis) and Oil Red O staining (for adipogenesis) (**Figure 1D**).

LGMSC-Exos were isolated and characterized by NTA, TEM observation, and western blotting analysis. As shown in **Figures 1E, F**, morphologically, LGMSC-Exos were spherical and cup-shaped, and NTA particle size analysis showed that they had the expected size of exosomes. Upon western blotting, we observed that isolated exosomes contained CD9, CD63 and Psg101, but had no calnexin (**Figure 1G**). Thus, the isolated vesicles exhibited characteristic morphologies and phenotypes of exosomes, confirming their successful isolation from the supernatants of cultured LGMSCs.

### LGMSC-Exos Treatment of NOD Mice Alleviated SS−Like Symptoms

**Figure 2:**
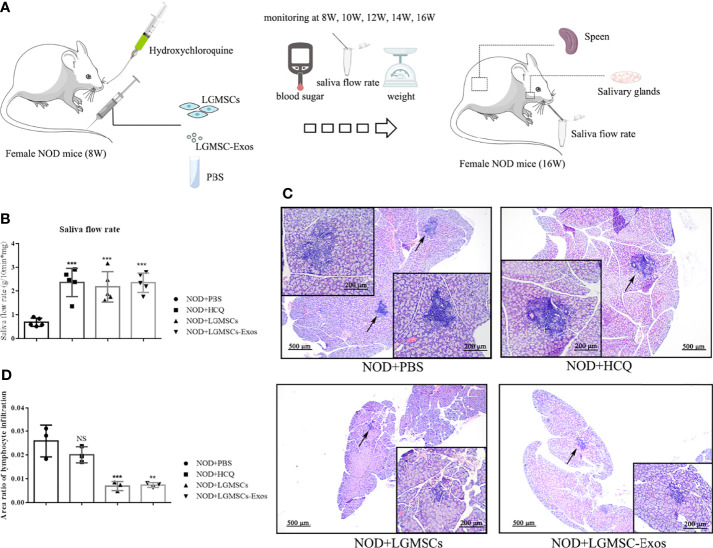
Transfer of LGMSC-Exos alleviates disease progression of NOD mice. **(A)** Schematic diagram of mice treatments; **(B)** Mouse saliva flow rate in the various groups. Data represent the mean ± standard deviation (SD; n = 3 independent experiments). ***p < 0.001, compared with the PBS group; **(C)** Representative histological images in the salivary gland sections of the various groups of stained using H&E (magnification: 100 × and 200 ×); **(D)** Area ratios of lymphocytic infiltration in the various groups. Data represent the mean ± standard deviation (SD; n = 3 independent experiments). NS, not significant, ***p < 0.001, **p < 0.01, compared with the PBS group.

LGMSC-Exos were injected into NOD mice *via* their tail veins. The therapeutic potential of LGMSC-Exos was evaluated using saliva flow rates and lymphocytic infiltration of submandibular glands of the NOD mice (**Figure 2A**). Remarkably, treatment with LGMSC-Exos improved the reduced saliva flow rates in the NOD mice significantly (**Figure 2B**). Furthermore, histopathological assessment of the LGMSC-Exo-treated NOD mice showed a considerably reduced area and number of lymphocyte infiltration foci compared with that in the PBS-treated NOD mice (p < 0.05, **Figures 2C, D**). The saliva flow rates and H&E staining both showed similar therapeutic effects of LGMSC-Exos compared with those of LGMSCs.

### LGMSC-Exo Administration Inhibited the Plasma Cell Response in NOD Mice

**Figure 3:**
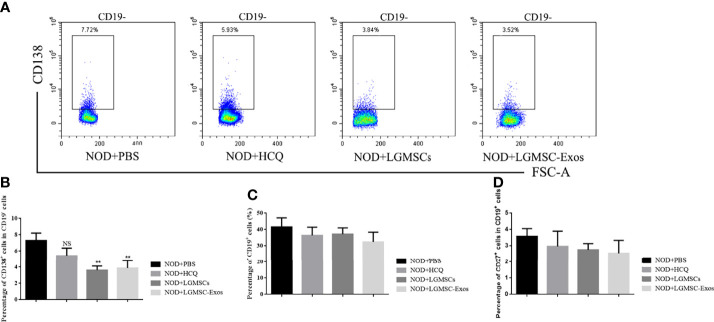
Transfer of LGMSC-Exo-altered B cell subsets of lymphocytes into the spleen of mice. **(A)** Representative flow cytometry profiles of CD3^-^CD19^-^CD138^+^ plasma cells from NOD mice after different treatments; **(B)** Percentages of CD138^+^ cells among CD19^-^ cells. Data represent the mean ± standard deviation (SD; n = 3 independent experiments). NS not significant, **p < 0.01, in comparison with the PBS group; **(C, D)** Percentages of CD19^+^ cells **(C)** and CD27^+^ cells among CD19^+^ cells **(D)**.

Splenic lymphocytes were collected and CD19^+^ B cells, CD19^+^CD27^+^ memory B cells, and CD19^-^CD138^+^ plasma cells were analyzed by flow cytometry. Compared with those in the PBS-treated group, the proportions of CD19^-^CD138^+^ plasma cells were significantly reduced in spleens of LGMSC-Exo-treated NOD mice (**Figures 3A, B**). However, the numbers of total B cells and memory B cells were not altered (**Figures 3C, D**).

### Effects of LGMSC-Exos on B Cell Subsets in the Co−Culture System

**Figure 4:**
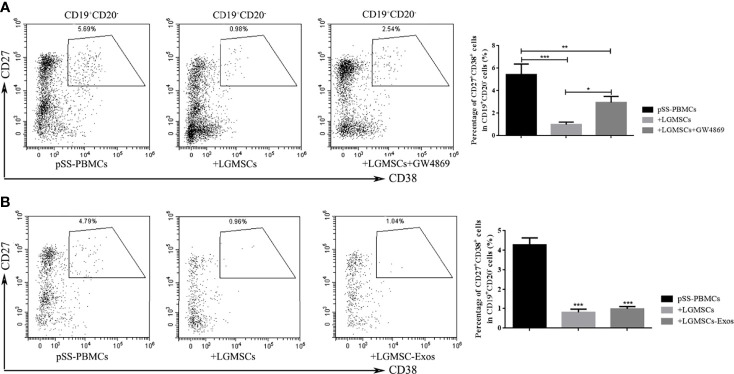
LGMSC-Exos inhibited the percentage of CD19^+^CD20^-^CD27^+^CD38^+^ plasma cells in the co−culture system *in vitro*. **(A)** The proportion of CD19^+^CD20^-^CD27^+^CD38^+^ plasma cells in PBMCs from patients with pSS co-cultured with LGMSCs and treated LGMSCs treated with GW4869. Data represent the mean ± standard deviation (SD; n = 3 independent experiments). ***p < 0.001, **p < 0.01, *p < 0.05. **(B)** The proportion of CD19^+^CD20^-^CD27^+^CD38^+^ plasma cells among PBMCs from patients with pSS co-cultured with LGMSCs and LGMSC-Exos. Data represent the mean ± standard deviation (SD; n = 3 independent experiments). ***p < 0.001, in comparison with the untreated group.

The LGMSC-Exos had a similar treatment effect in NOD mice compared with their parental cells. To investigate whether the effect on plasma cells was mediated, or at least partially mediated, by exosomes, we evaluated the effect *in vitro*. As shown in **Figure 4A**, when LGMSCs were treated with the exosome inhibitor GW4869, their effect on plasma cells was partially abolished. Moreover, when PBMCs from patients with pSS were cocultured with LGMSC-Exos, we observed a markedly decreased in the percentage of CD19^+^CD20^-^CD24^+^CD38^+^ plasma cells (**Figure 4B**), showing similar effects to their parental cells.

### *PRDM1* Was Regulated by LGMSC-Exos in the Co−Culture System

**Figure 5:**
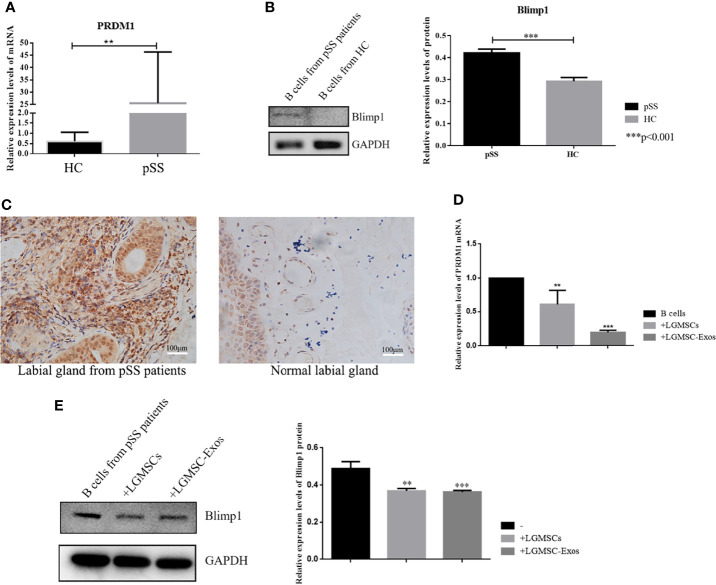
PRDM1 expression is regulated by LGMSC-Exos. **(A)** Relative mRNA expression levels of *PRDM1* in B cells from HCs and patients with pSS. **(B)** Results of western blotting for PRDM1 in B cells from HCs and patients with pSS. **(C)** Immunohistochemical staining of PRDM1 in the labial glands from patients with pSS and normal labial glands. **(D)** Relative mRNA expression levels of *PRDM1* after coculture with LGMSCs and LGMSC-Exos. **(E)** Western blotting analysis of PRDM1 after coculture with LGMSCs and LGMSC-Exos. Data represent the mean ± standard deviation (SD; n = 3 independent experiments). ***p < 0.001, **p < 0.01.

To identify the likely target *via* which LGMSC-Exos mediate their inhibitory effect on plasma cells, mRNA profiling of B-lymphocytes activated in the presence or absence of exosomes was searched in the literature (24). PRDM1, also known as B lymphocyte induced maturation protein (Blimp1), is a regulator of B cell differentiation. According to mRNA profiling data from Khare et al., *PRDM1* was one of the differentially expressed genes. Thus, we investigated whether the expression of *PRDM1* in PBMCs from patients with pSS was regulated by LGMSC-Exos. Firstly, we detected the mRNA and protein levels of PRDM1/Blimp1 in B cells from pSS patients. As shown in **Figures 5A, B**, PRDM1 expression was increased in B cells from patients with pSS compared with that in B cells from healthy controls (HCs). Moreover, immunohistochemical staining showed increased levels of PRDM1 in labial glands from patients with pSS (**Figure 5C**). Furthermore, 72 hours after coculture, PBMCs were collected and CD19^+^ B cells were purified for PCR and western blotting analysis. The results showed that both the mRNA level and protein levels of PRDM1 were reduced after co-culture with LGMSC-Exos (**Figures 5D, E**).

### MicroRNAs Carried by LGMSC-Exos

**Figure 6:**
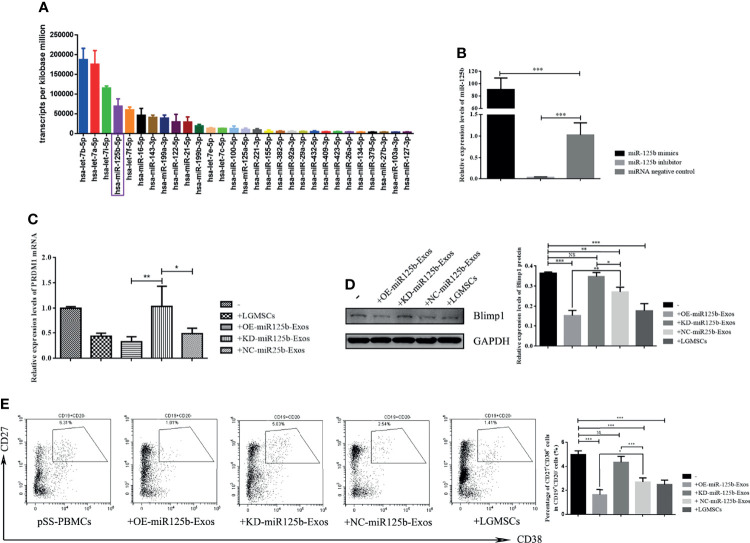
MiR-125b mediates LGMSC-Exos-induced plasma cell alteration and *PRDM1* expression. **(A)** Top thirty most abundant miRNAs in LGMSC-Exos, as assessed by miRNA sequencing. **(B)** qRT-PCR results of the knockdown and overexpression efficiency after transfection with miR-125b mimics and inhibitors. **(C)** Relative mRNA expression levels of *PRDM1* after coculture with transfected LGMSC-Exos. **(D)** Western blotting analysis of PRDM1 after cocultured with transfected LGMSC-Exos. **(E)** Representative flow cytometry profiles and statistical results of the proportions of CD19^+^CD20^-^CD27^+^CD38^+^ plasma cells. Data represent the mean ± standard deviation (SD; n = 3 independent experiments). NS, not significant, ***p < 0.001, **p < 0.01, *p < 0.05.

To identify exosomal miRNAs that might exert the therapeutic effect of LGMSC-Exos, we furthered examined the miRNAs carried by LGMSC-Exos using miRNA sequencing. The top 30 most abundant miRNAs in LGMSC-Exos are shown in **Figure 6A**. Among those miRNAs, has-miR-125b-5p (miR-125b) was chosen for further analysis after a literature review (25). Overall, LGMSC-Exos carry approximately 462 miRNAs, including 65 novel miRNAs (**Supplementary Table S1**).

### miR-125b Mediates LGMSC-Exos-Induced CD19^+^CD20^-^CD24^+^CD38^+^ Plasma Cell Alteration and *PRDM1* Expression

MiR-125b was overexpressed (OE) or knocked down (KD) in LGMSC-Exos by transfection of miR-125b mimics or inhibitors, respectively. As shown in **Figure 6B**, the transfection efficiency was demonstrated by qRT-PCR. PBMCs from patients with pSS were co-cultured with OE-miR125b-Exos or KD-miR125b-Exos, followed by flow cytometry detection of B cell subset percentages in the collected PBMCs, accompanied by western blotting and qRT-PCR detection of PRDM1 expression. The results showed that OE-miR125b-Exos treatment slightly reduced the mRNA levels of *PRDM1* whereas the mRNA levels of *PRDM1* was significantly enhanced after KD-miR125b-Exos treatment (**Figure 6C**). These results were confirmed by western blotting (**Figure 6D**). Furthermore, the percentage of CD19^+^CD20^-^CD24^+^CD38^+^ plasma cells was significantly reduced in OE-miR125b-Exos treatment group, whereas KD-miR125b-Exos treatment had the opposite effect (**Figure 6E**).

### miR125b Directly Targets *PRDM1*

**Figure 7:**
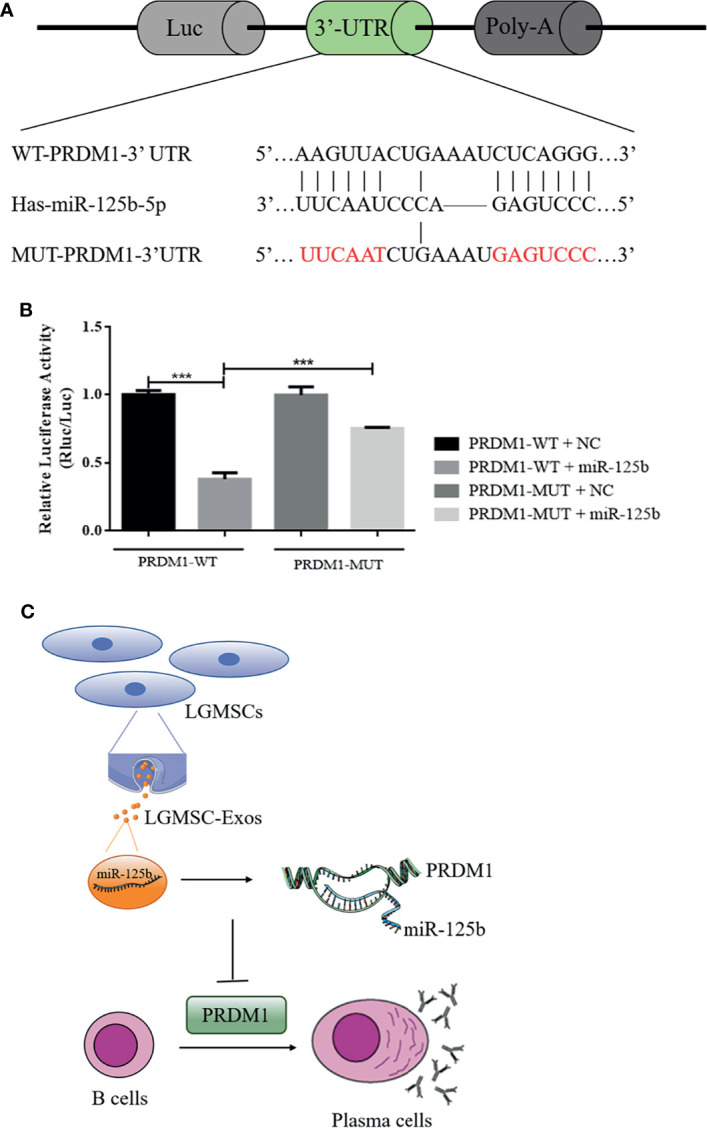
*PRDM1* is a direct target of miR-125b and the putative mechanism of LGMSC-Exos on plasma cells. **(A)** The seed sequence of miR-125b located in the *PRDM1* wild-type 3′-UTR and the mutated sequence. **(B)** Luciferase activities after co-transfection of miR-125b and the *PRDM1* wild-type 3′-UTR and mutant 3′-UTR. Data represent the mean ± standard deviation (SD; n = 3 independent experiments). ***p < 0.001. **(C)** Putative mechanism of LGMSC-Exos on plasma cells involving miR-125b and PRDM1.

To determine whether miR-125b targets potential sites in *PRDM1*, dual luciferase reporter assays were performed (**Figure 7A**), which demonstrated a significant reduction in the relative luciferase activity from the PRDM1–WT plasmid compared with that from the PRDM1–MUT vector in the presence of miR-125b mimics (**Figure 7B**). Thus, miR-125b targets the 3′-UTR of *PRDM1* to restrain its expression.

## Discussion

At present, no available drug can cure pSS. However, studies indicate that MSCs might represent a promising strategy to treat pSS (26, 27). Therapies based on MSCs have the potential to reduce inflammation and preserve salivary function in patients with pSS (28). Exosomes have similar effects to their parental MSCs, transporting a variety of molecules to change the activity of the recipient cells. It is easier to store exosomes than MSCs, with none risks associated with cell transplantation (29). To study cell-free treatment based on MSC-derived exosomes, LGMSC-Exos were evaluated to treat pSS and determine the potential mechanism. The results showed that exosomes derived from LGMSCs are as effective as their parental cells in treating experimental pSS, suggesting that exosomes derived from LGMSCs might hold promise to treat pSS and provide a new therapeutic strategy for autoimmune diseases.

The last few decades have witnessed the discovery of a variety of B-lymphocyte populations (30). Recent data have further demonstrated that B cell subsets and their activation contribute to autoimmune disease (31). The importance of B cells in pSS pathogenesis has been reported; however, which B-cell subset(s) underlie the autoimmune features of pSS, and might respond to treatment, remain unknown (32). In this study, our flow cytometry analysis could detect the percentages of CD19^+^CD20^-^CD27^+^CD38^+^ plasma cells, CD19^+^CD24^high^CD38^high^ transitional B cells, CD19^+^CD24^hi^CD27^+^ B regulatory cells, CD19^+^IgD^+^CD38^high^ plasmablasts, CD19^+^CD27^-^IgD^-^ double negative B cells, CD19^+^CD27^-^IgD^+^ naïve B cells, CD19^+^CD27^+^ total memory B cells, CD19^+^CD20^-^ B cells, and CD19^+^ total B cells, to find out which subsets of B lymphocytes are affected by LGMSCs or LGMSCs-Exos. The results of *in vitro* and *in vivo* experiments were consistent, showing that plasma cell proportions were decreased after treatment with LGMSCs and their exosomes. In patients with pSS, these plasma cells are increased and correlate positively with autoantibody positivity, disease activity, and serum IgG levels (33). Moreover, up to 50% of infiltrating B cells in the salivary glands of patients with pSS are fully differentiated plasma cells. These findings indicate the necessity of targeting plasma cells in pSS. Remarkably, LGMSCs and their exosomes targeted plasma cells in the NOD mice and PBMCs from patients with pSS, providing novel insights and targets for pSS treatment.

A large scale genome-wide association study revealed the involvement of certain genetic loci in pSS. *PRDM1*, encoding a transcription factor that is important in plasma cell differentiation, was one of the regions discovered to be involved in pSS (34). Zhang et al. emphasized this pSS‐associated gene and suggested that *PRDM1* was significantly upregulated in patients with pSS and exhibited increased expression during pSS pathogenesis (35). The present study confirmed that *PRDM1* was unregulated in both PBMCs and the labial glands of patients with pSS. According to the mRNA profiling of B-lymphocytes incubated with or without exosomes, *PRDM1* was one of the differentially expressed genes (24). Therefore, the present study further explored *PRDM1* as target of LGMSC-Exos. After treatment with LGMSC-Exos, the expression of *PRDM1* decreased significantly, suggesting that it could be a target of MSCs-Exos to treat pSS. These findings might support a future study of PRDM1 as a focus in pSS pathogenesis research or in MSC-Exo-based therapy.

Exosomes contain a wide variety of molecules, including proteins, lipids, DNAs, mRNAs, and miRNAs. Gene-based communication among mammalian cells is based on the transfer of exosome-carried unique miRNAs or novel mRNAs to recipient cells (36, 37). MiRNAs have attracted the most attention because of their regulatory effects on gene expression (38). Therefore, miRNA array profiling was used to identify LGMSC-Exo-carried miRNAs, which identified approximately 462 miRNAs (**Supplementary Table S1**). Among the top 10 most abundant miRNAs, miR-125b attracted our attention. MiR-125b plays important roles in hematopoiesis and immune cell function (25). Overexpression of miR-125b inhibited plasma cell differentiation (39, 40), and it is predicted to be bind to *PRDM1*. Thus, it was selected for further investigation. The results demonstrated that miR-125b binds directly to *PRDM1* and depletion or upregulation miR-125b in LGMSC-Exos had a significant impact on the inhibitory effects of LGMSC-Exos on plasma cell differentiation and *PRDM1* expression. These findings revealed the partial mechanism by which LGMSC-Exos inhibit plasma cells and might provide new ideas to enhance the therapeutic function of exosomes to treat autoimmune diseases like pSS.

In summary, compared to their parental cells, LGMSCs-Exos exhibited comparable effects in reducing inflammatory infiltration and restoring salivary gland secretory function in NOD mice. Moreover, *in vitro*, they showed similar inhibitory effects on CD19^+^CD20^-^CD27^+^CD38^+^ plasma cells to LGMSCs. The underlying mechanism by which LGMSC-Exos inhibit the differentiation of plasma cells involves exosomal miR-125b binding to and inhibiting *PRDM1* mRNA translation (**Figure 7C**). The present study suggested that LGMSC-Exos represent a possible new cell-free therapy to treat B cell-related inflammatory diseases and indicated that the miR-125b/PRDM1 axis mediates plasma cells inhibition, which might open up new avenues for potential therapeutic targets in pSS.

## Data Availability Statement

The original contributions presented in the study are included in the article/**Supplementary Material**. Further inquiries can be directed to the corresponding authors.

## Ethics Statement

The studies involving human participants were reviewed and approved by Institutional review board of Peking University School of Stomatology. The patients/participants provided their written informed consent to participate in this study. The animal study was reviewed and approved by Institutional review board of Peking University School of Stomatology.

## Author Contributions

YX performed the experiments, analyzed the data, and drafted the manuscript. BL performed the experiments. JH and HH designed the study and revised the manuscript. All authors contributed to the article and approved the submitted version.

## Funding

This work was supported by the National Natural Science Foundation of China (grant number 81970952).

## Conflict of Interest

The authors declare that the research was conducted in the absence of any commercial or financial relationships that could be construed as a potential conflict of interest.

## Publisher’s Note

All claims expressed in this article are solely those of the authors and do not necessarily represent those of their affiliated organizations, or those of the publisher, the editors and the reviewers. Any product that may be evaluated in this article, or claim that may be made by its manufacturer, is not guaranteed or endorsed by the publisher.
